# Performance Assessment of Mechanically Recycled EPS

**DOI:** 10.3390/ma18194547

**Published:** 2025-09-30

**Authors:** Domagoj Tkalčić, Jelena Vukadin, Bojan Milovanović, Ivana Banjad Pečur

**Affiliations:** Faculty of Civil Engineering, University of Zagreb, 10000 Zagreb, Croatia; jelena.vukadin@grad.unizg.hr (J.V.); bojan.milovanovic@grad.unizg.hr (B.M.); ivana.banjad.pecur@grad.unizg.hr (I.B.P.)

**Keywords:** expanded polystyrene, virgin EPS, recycled EPS, mechanical properties, thermal properties, density

## Abstract

This study investigates the influence of mechanically recycled EPS on the mechanical and thermal properties of EPS composites for use in thermal insulation. Composites containing 5%, 10%, 25%, and 50% recycled EPS were produced using two recycled sources (construction EPS and packaging EPS). The tested properties included density, compressive strength at 10% deformation, bending strength, and thermal conductivity. Results show that increasing recycled content leads to a decline in density, with a more pronounced drop at higher recycled levels, particularly above 10% recycled content in the S1–25 series and 25% in the other series. Compressive strength correlates closely with density regardless of recycled content or origin and the behavior largely aligns with EN 13163 predictive models for virgin EPS. Thermal conductivity remains unchanged at lower recycled contents, with minor increases (up to 6%) observed at 50% recycled content. Bending strength decreases with increasing recycled content with greater losses noted in specimens containing packaging EPS. However, despite containing recycled materials, the empirical equations stated in EN 13163:2016 for predicting thermal conductivity and compressive stress at 10% deformation remain closely correlated regardless of recycled material content, indicating that density remains the main parameter for predicting the tested properties. These findings suggest that EPS with moderate recycled content can meet performance expectations, though further research on microstructure is recommended to understand property degradation mechanisms at higher recycled levels.

## 1. Introduction

The future of the built environment in Europe is defined by the need to deliver higher standards of energy efficiency, indoor comfort, and sustainability in response to evolving regulatory frameworks and societal expectations. Many existing buildings, particularly those constructed before the early 2000s, fall short of these requirements and require significant upgrades to meet modern performance standards. These buildings, while no longer optimal in terms of thermal performance, continue to provide valuable residential and functional space and are essential to the continuity of urban life. Full-scale demolition of such structures is rarely a viable option, as it would disrupt the urban infrastructure and social fabric. Instead, energy refurbishment offers a path to extending their service life while reducing energy demand and improving indoor environmental quality [1,2]. In parallel, there has been a growing shift toward sustainability in the built environment, driven by climate goals, circular economy principles, and increased awareness of resource efficiency. This transition emphasizes the use of recycled materials and products containing recycled content to minimize environmental impacts and reduce dependence on virgin raw materials. Integrating such materials into construction and renovation projects is increasingly viewed as a key strategy for reducing embodied carbon and promoting sustainable building practices [3,4,5].

On the other hand, polymer-based materials play an identified role in everyday life due to their favorable properties. In the construction sector, expanded polystyrene (EPS) is primarily used as thermal insulation for building envelopes to meet requirements prescribed by laws, regulations, and European directives.

According to 2023 data, 413.8 Mt of plastics are produced annually worldwide, an increase of 43.1 Mt compared with 2018 [6]. EPS, which is known for its thermal insulation and shock-absorbing properties, represents a significant share of this total. EPS continues to play a significant role in the packaging industry. In 2023, global EPS production exceeded 7.3 Mt, with more than 2.6 Mt allocated to packaging applications. Europe alone consumed around 1.1 Mt of EPS for packaging that year. Packaging, including food containers, appliance protection, and electronics cushioning, accounts for approximately 40% of global EPS consumption [7].

In 2025, the European EPS market is projected to reach 2.2 Mt, with growth expected to continue toward 2.62 Mt by 2030 [8]. EUMEPS [9] indicates that approximately 75% of European EPS production is dedicated to the construction industry, largely for building insulation applications aimed at improving energy performance. This includes the widespread use of external thermal insulation composite systems (ETICS), which have become a standard retrofit and new build solution across Europe. In 2017, over 234 million m^2^ of external walls were insulated using ETICS. By 2020, this figure rose to approximately 332 million m^2^, with an average insulation thickness of around 125 mm [10]. It is estimated that ETICSs account for roughly 70% of all EPS-based insulation installations [11].

While EPS has played a vital role in improving building energy efficiency, a major challenge arises as the materials installed in facades from the 1970s through the early 2000s approach their functional service life. These aging insulation systems, many of which no longer meet current regulatory standards for thermal performance due to aging and low thickness, must now be removed and replaced. This results in the generation of substantial volumes of EPS waste, for which Europe currently lacks a harmonized and sustainable recycling solution. Current disposal practices, such as landfilling and incinerations, are increasingly at odds with circular economic principles and environmental policy targets [1]. Currently, polymer waste is managed in several ways, including incineration, landfilling, and recycling. Polymer materials are often not biodegradable, which is why they most commonly end up in landfills or are energetically recovered through incineration, but this method of polymer waste management is not considered an environmentally friendly solution [12,13,14]. Therefore, developing effective recycling and reuse strategies for this material has become an urgent priority.

Mechanical and chemical recycling are the two main approaches when it comes to polymer/expanded polystyrene (EPS) recycling technology [15]. Mechanical recycling is based on crushing or grinding waste EPS to obtain smaller bead aggregates, which are then used as a substitute for EPS. For example, EPS waste can be mechanically ground into tiny beads or larger granules. According to Eurostat, the European Union recycled 40.7% of plastic packaging in 2023 [16]. Mechanical recycling of plastic waste in Europe in 2022 amounted to 12.6% (6.8 Mt) [6]. If these data are compared with plastic mechanical recycling data from 2018, an increase of as much as 70% can be observed, clearly demonstrating the recycling trend. Mechanical recycling of polypropylene (PP) [17], polyethylene (PE) [18], and polyvinyl chloride (PVC) [19,20] is already a developed and accepted method, while polystyrene (PS) recycling is poorly represented in the literature.

Several studies have been published on mechanically recycled EPS. A study on EPS with 40% mechanically recycled EPS from 2017 [21] claims that there was no significant loss of mechanical and thermal properties. The authors claim that the performance of EPS with recycled material can be predicted by the same empirical equations used for virgin EPS and that the properties depend solely on the material’s density.

Acierno et al. [22] present results from tested samples with 10, 15, and 20% mechanically recycled EPS. The study focuses on thermal conductivity and the results show that reference samples with 0% recycled content showed an 18% increase in thermal conductivity compared with the virgin reference samples. According to the authors, samples with 20% recycled content exhibited thermal conductivity values that were 5% lower than those of the reference material. It must be said that these results did not yield any clear conclusions, and further testing needs to be done.

Recent work by Gaši et al. (2022) [23] demonstrated that even minor modifications, such as perforations and slits, can significantly improve virgin EPS’s hydrothermal behavior, increasing vapor diffusion by up to 42% while causing only a minor (~9%) increase in thermal conductivity. Their findings underscore the importance of understanding how structural changes affect both moisture transport and thermal performance. In this context, the impact of mechanical recycling, which inherently alters the material’s internal structure through grinding and reprocessing, becomes particularly relevant. With the emerging challenge of managing large quantities of EPS that require recycling, there is a clear and growing need to develop sustainable solutions for EPS recycling. While chemical recycling processes can transform it back into polystyrene, and some studies have explored extending its service life by incorporating it into concrete or mortars, the vast majority of EPS waste is still disposed of through landfilling or incineration. Notably, the body of research on mechanical recycling of EPS remains limited, and the available studies often report contradictory or inconclusive results. In response to this research gap, the present study offers a comprehensive investigation into the mechanical and thermal performance of EPS materials incorporating mechanically recycled content. The results presented in this paper provide new, structured insights into how recycled EPS affects key material properties, contributing to a better understanding of its potential reuse in thermal insulation applications.

This research presented in this paper was built upon previous preliminary studies by the authors, in which the mechanical properties of EPS with mechanically recycled content were introduced and briefly analyzed [24,25]. These initial findings, presented in the form of conference papers, provided a general overview of the effects of recycled EPS on selected material characteristics. The present work expands significantly on those contributions.

## 2. Materials and Methods

### Material Description

Two different densities of EPS were targeted in this study in the production process for all EPS samples (nominal densities of 20 and 25 kg/m^3^). The performance of samples containing a certain percentage of mechanically recycled EPS was compared to that of the virgin material, i.e., reference samples containing EPS with 0% recycled content.

**Figure 1:**
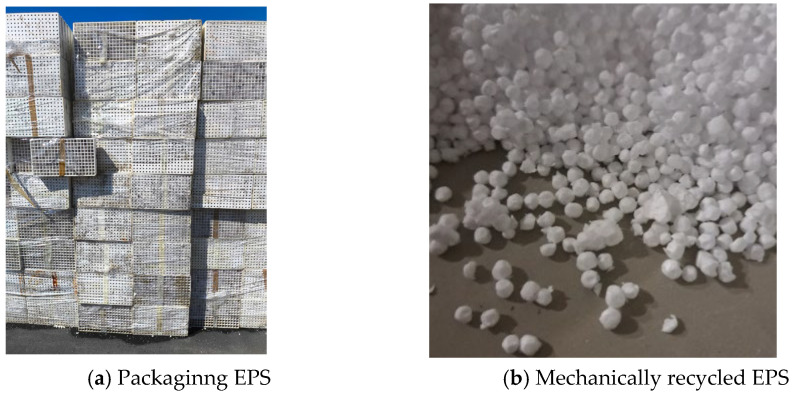
Mechanical recycling.

EPS from two different sources was mechanically recycled (construction EPS (S1), originally in the form of boards, and agricultural packaging EPS (S2), originally in the form of trays). To achieve maximum homogeneity between the virgin and recycled EPS, both types of used EPS were milled into smaller particles prior to reuse (Figure 1).

The construction EPS used in this study was visually clean and free of significant contamination or surface impurities, while the impurities in the packaging EPS were separated in the grinding process.

The average bulk density of both recycled materials (S1 and S2) was in the range of 15–17 kg/m^3^.

Virgin EPS was swapped for recycled EPS by volume (5%, 10%, 25%, and 50%). The recycled content in each block is further explained in Table 1. As a clarification for labels, S1-20-5% means construction EPS with density of 20 kg/m^3^ and 5% recycled EPS content by volume.

**Figure 2:**
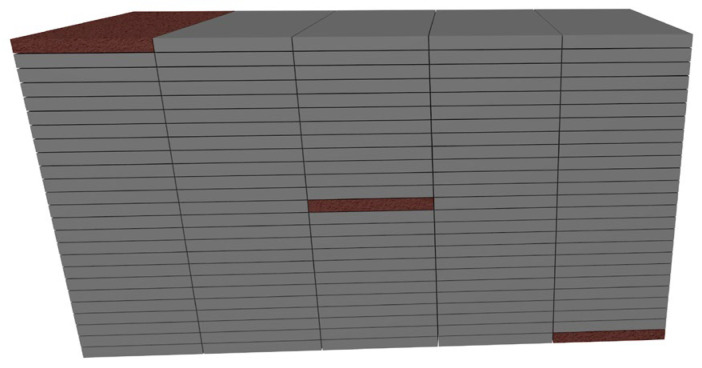
Board location in the block.

A total of 18 unique EPS blocks were produced, each with nominal dimensions of 2600 × 1300 × 1050 mm. From each of the blocks, 3 boards measuring 500 × 1000 × 50 mm were extracted along the diagonal plane using a hot wire cutting technique. The selected boards, visually distinguished in Figure 2, represent defined positions within the block to ensure traceability of the sample location and enable analysis of representative samples, taking into account the spatial property variability within the EPS block.

In order to establish the differences in performance between virgin EPS and recycled EPS, the following tests were performed (Table 2).

**Figure 3:**
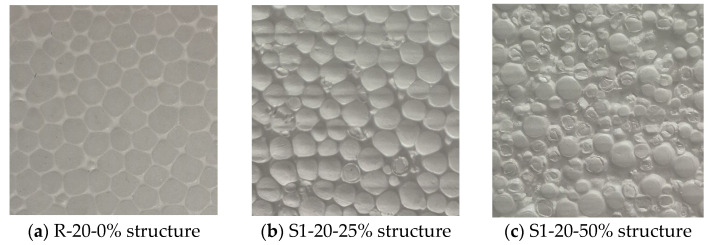
Structure of different EPS series.

Differences between series with various recycled contents can be observed with the naked eye. Figure 3 shows the structure of 3 different blocks. All 3 samples have a nominal density of 20 kg/m^3^.

**Figure 4:**
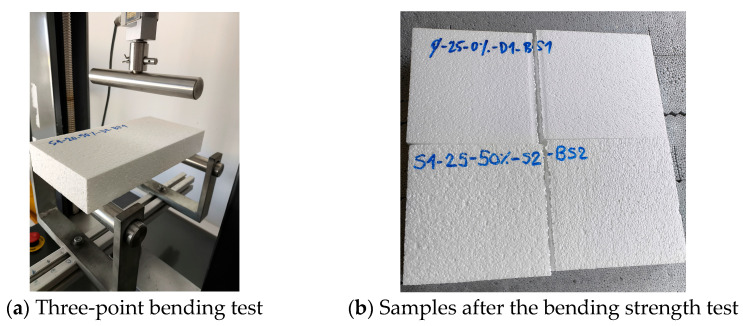
Three-point bending test setup and broken samples.

Figure 4 shows the bending strength test setup and broken samples. All mechanical properties were determined on the same hydraulic press but with different tools.

The apparent density ( $\rho_{a}$ ) of the EPS samples was determined following the specification of EN ISO 29470:2020 [26]. Rectangular test specimens cut from blocks with hot wire were accurately weighted using a Kern PCD10KO non-automatic electronic precision balance scale. Their precise dimensions (length, width, and thickness) were subsequently measured with a Stiro-Lab XYZ.320 measuring table (Stirolab, Logatec, Slovenia) and a Suki 0–150 mm caliper (suki.international GmbH, Landscheid, Germany).

A universal testing machine (Excel Didactic, model FS.10, Siemens, Munich, Germany), equipped with a 10 kN force measurement cell, was utilized to assess the mechanical performance of the EPS.

Compressive strength at 10% deformation ( $\sigma_{10}$ ) tests were conducted on 100 × 100 × 50 mm rectangular samples. Each specimen was subjected to a preload of 250 Pa before being compressed at a constant rate of 10 mm/min. This procedure strictly adhered to the requirement of the EN ISO 29469:2022 standard [27]. To ensure representative data and account for potential variations within the material blocks, fifteen specimens were tested per block, with samples taken equally from the top, middle, and bottom layers.

To determine bending strength ( $\sigma_{bs}$ ), 3-point bending tests were performed. Rectangular samples measuring 300 × 150 × 50 mm were used. These specimens were tested with a supporting span of 250 mm and a loading speed of 10 mm/min. The testing methodology conformed to Method B of the EN 12089:2013 standard [28]. A total of nine bending specimens were tested per block, also sampled equally from the top, middle, and bottom layers.

The thermal conductivity ( λ ) of the EPS samples was measured using a Stiro-Lab LM PLUS 305 heat flow meter (Stirolab, Logatec, Slovenia). Samples with dimensions of 300 × 300 × 50 mm were prepared for this test. Measurements were performed in accordance with EN 12667:2002 [29], maintaining a mean temperature of 10 °C and a temperature difference of 10 °C between the hot and cold plates during the test.

## 3. Results

**Figure 5:**
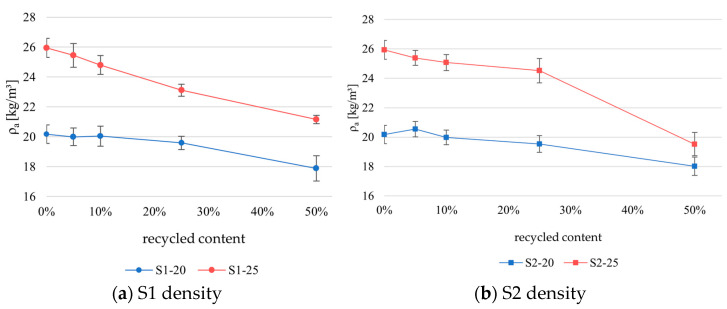
Density of S1 and S2.

Figure 5 shows the variation in density across the samples, categorized by their percentage of recycled content measured according to EN ISO 29470:2020 [26].

The results clearly show a drop in density with an increase in the recycled EPS volume due to the lower recycled EPS density. As expected, the density drop is more pronounced in samples with a higher density of virgin EPS. S1–25 shows a steady degradation in density as the recycled content increases. However, it should be noted that the significant drop in density is more pronounced when the recycled EPS content is higher than 25% for the other series. S1–20 and S2–20 tolerate recycled content up to ~25% without a major density loss; beyond that, it becomes less consistent. The drop in density with an increase in recycled material content is expected, as recycled EPS has lower density than the virgin material. The standard deviation in the density measurements remained relatively stable and was comparable to the reference materials.

**Figure 6:**
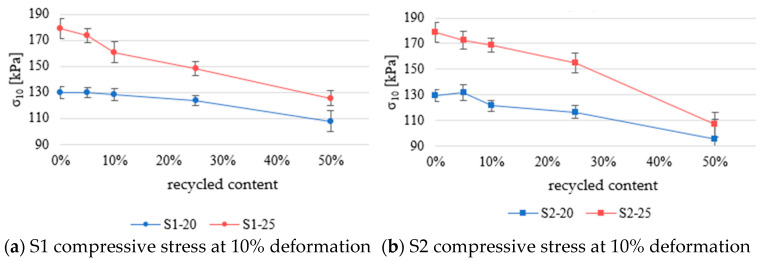
Compressive stress of S1 and S2.

Figure 6 shows the compressive stress at 10% deformation of samples measured according to EN ISO 29469:2022 [27]. The results are categorized by type and percentage of recycled content.

When examining the compressive stress at 10% deformation results (Figure 6a), the S1-20 series samples containing 50% recycled EPS show values that are 17% lower compared with the reference samples.

Among the S2–20 series (Figure 6b), samples with 50% recycled EPS had 27% lower values than the reference samples.

The compressive strength results show consistent standard deviation values.

Series S1–25 and S2–25 have a more significant performance drop with increased recycled content, which is expected due to the pronounced drop in density. According to EN 13163:2016 [30], compressive stress at 10% deformation depends only on the density of the EPS. Even though the empirical equations set out in EN 13163:2016 are used for determining the compressive strength at 10% deformation of virgin material, the results obtained in this study indicate that they can be used to predict the properties of EPS with mechanically recycled EPS.

(1) $$\sigma_{10,\;mean}=10.0\;kPa\cdot \frac{m^{3}}{kg}\times \rho_{a}-81.0\;kPa\;[kPa]$$

(2) $$\sigma_{10,\;pred}\approx 10.0\;kPa\cdot \frac{m^{3}}{kg}\times \rho_{a}-109.1\;kPa\;[kPa]$$

Equation (1) enables indirect estimation of $\sigma_{10}$ for EPS products based on known density and is derived from a large dataset collected across European manufacturing facilities for virgin EPS. Equation (2) defines the lower limit of the 95% confidence interval associated with that relationship.

**Figure 7:**
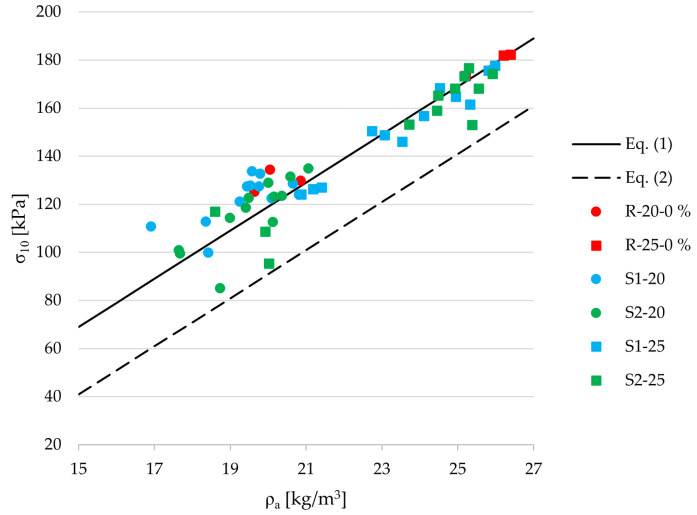
$\sigma_{10}$ approximation according to EN ISO 13163:2016.

Figure 7 presents the mean values of compressive strength at 10% deformation for samples taken from the bottom, middle, and top sections of each EPS block, across all recycled content levels for both types of recycled EPS.

The data points show a consistent alignment along the linear trend defined by Equation (1), which originates from the empirical relationship between compressive strength and density for virgin EPS, as specified in European standards. This close clustering—regardless of the recycled content or its type—suggests that the mechanical performance of the tested materials remains within the expected tolerance, even when incorporating up to 50% recycled EPS. Notably, this includes samples containing recycled packaging EPS, which also follow the predictive trend with a reasonable degree of accuracy. Only a small number of samples fall near the secondary curve representing the lower bound of the 95% confidence interval, indicating that the majority of specimens meet the compressive strength at 10% deformation performance expectations for standard compliant EPS. These findings support the validity of applying existing predictive models developed for virgin EPS to materials that include significant proportions of recycled EPS.

To evaluate whether the empirical model for compressive stress at 10% deformation defined in EN 13163:2016 remains valid for EPS containing recycled content, the coefficient of determination ( $R^{2}$ ) was calculated for each material series. This comparison assesses how well the standard model fits the experimental data obtained from EPS composites with varying amounts of mechanically recycled material. The results are presented in Table 3.

All calculated $R^{2}$ values indicate a strong correlation between the experimental data and the EN 13163:2016 model, with the lowest value being 0.962 for the S2-25-50% series. This high level of agreement suggests that the standard model can be reliably used to predict compressive strength at 10% deformation for EPS materials containing up to 50% recycled content.

This outcome implies that density remains the dominant predictor of compressive performance, while microstructural imperfections introduced through recycling, such as bead fragmentation or surface irregularities, may not sufficiently severely disrupt the overall mechanical integrity. It also suggests that the interfacial bonding between virgin and recycled EPS beads is adequately preserved during the molding process, enabling effective stress transfer through the material. As a result, density remains the best indicator of the key structural parameter predicting compressive strength, while potential microstructural degradation does not appear to manifest in a way that significantly alters the macroscopic mechanical behavior.

**Figure 8:**
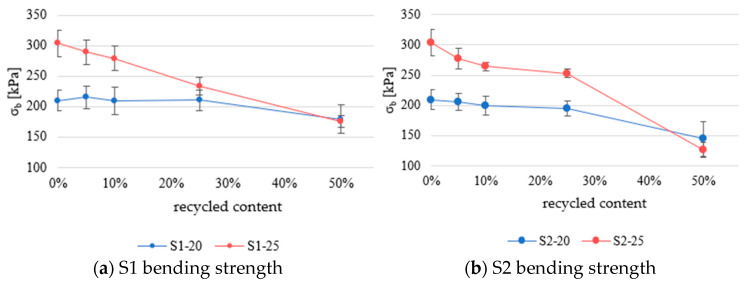
Bending strength of S1 and S2.

Figure 8 shows the results for bending strength. The results are categorized by type and percentage of recycled content. EN 13163:2016 does not recommend empirical equations for predicting bending strength based on density.

Samples containing recycled packaging EPS (S2 series) exhibit less than a 5% difference in performance compared with samples with recycled construction EPS (S1 series) at equivalent recycled content levels, indicating no significant variation between the two sources until more than 25% recycled material is added.

The S1–25 series with 10% recycled material has almost 22% lower bending strength than the reference material. As the recycled material content increases, the bending strength continues its downward trend. Samples with 50% recycled material have 42.6% lower values than the reference material.

When comparing recycling materials, materials with recycled construction EPS (S1 series) show better retention of properties compared with material with recycled packaging EPS (S2 series).

**Figure 9:**
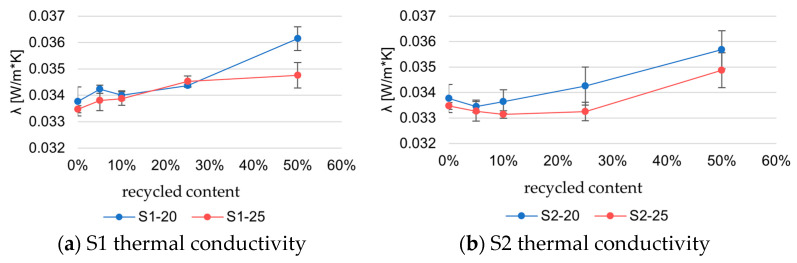
Thermal conductivity of S1 and S2.

Figure 9 presents the results on the thermal conductivity of samples. The results are categorized by origin and percentage of recycled content.

Series S1–20 and S1–25 exhibit less than a 5% change in thermal conductivity when the recycled EPS content is below 10%. The S1–25 series was expected to have lower thermal conductivity than the S1–20 series due to its higher density. The biggest difference between the reference material and the material with recycled EPS is shown in S1–20 at 50% recycled EPS content, where the samples with 50% recycled content have 6.6% higher thermal conductivity.

There is less than a 5% increase in thermal conductivity in samples S1–20 and S1–25 while the recycled EPS is below 25% when compared with the reference material, while the S1–25 series with 50% recycled material has an approximately 4% increase in thermal conductivity.

Similarly, the S2 series exhibits less than a 5% difference in thermal conductivity until the recycled content is increased above 25%. Samples with 50% recycled EPS have around a 6% increase (S2–20 series) and a 4% increase (S2–25 series).

Thermal conductivity measurements show low standard deviation values for samples up to 25% recycled content, indicating stable thermal performance. At 50% recycled EPS, slight increases in variability are observed, especially for the S2 series, but they remain within acceptable limits. The results suggest that, even at higher recycled fractions, the thermal behavior remains predictable.

**Figure 10:**
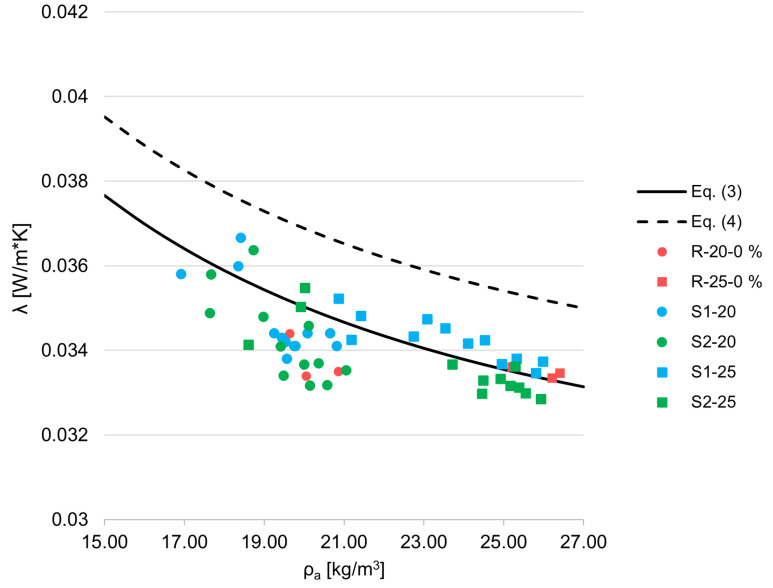
Comparison of EN ISO 13163:2016 and recycled EPS.

Figure 10 represents the relationship between thermal conductivity and apparent density. Equations (3) and (4) are taken from EN ISO 13163:2016 and represent curves for indirect testing of non-infrared-absorbing EPS.

(3) $$\lambda_{mean}=0.025314+5.1743\times 10^{-5}10-5\times \rho_{a}+\frac{0.173606}{\rho_{a}}$$

(4) $$\lambda_{pred}\approx 0.027174+5.1743\times 10^{-5}10-5\times \rho_{a}+\frac{0.173606}{\rho_{a}}$$

The reference samples (R-20-0% and R-25-0%) have thermal conductivity that is very close to or slightly below the curve represented by Equation (3), which represents the standard relationship between thermal conductivity and density for virgin EPS. Importantly, none of the measured results exceed the curve defined by Equation (4), which corresponds to the 95% confidence interval. This indicates that the thermal performance of these samples falls well within the expected variability for standard factory-produced EPS. Notably, the S2–20 series, which incorporates recycled EPS from packaging waste, displays thermal conductivity values comparable to those of the S1–20 series. This suggests that, at this level of recycled content, the source and prior use of the recycled material do not have a significant influence on the thermal conductivity of the final product. The dataset in this study focuses primarily on EPS materials with nominal densities in the range of 20–25 kg/m^3^. To fully characterize thermal conductivity behavior across the broader application spectrum, further validation is required for materials with nominal densities below 20 kg/m^3^ and higher than 25 kg/m^3^.

Similarly to the data for compressive stress at 10%, evaluation using the empirical model for thermal conductivity defined in EN 13163:2016 remains valid for EPS containing recycled content, and the coefficient of determination ( $R^{2}$ ) was calculated for each material series. This comparison assesses how well the standard model fits the experimental data obtained from EPS composites with varying amounts of mechanically recycled material. The results are presented in Table 4.

## 4. Discussion

One of the key factors governing the thermal performance of EPS is gas-phase conduction, which typically accounts for the largest share of total heat transfer in closed-cell foams. The extent of this conduction is not constant but rather influenced by the cell size and the mean free path of gas molecules. This interaction is described by the Knudsen effect, which becomes significant when the characteristic cell diameter approaches the molecular mean free path. In such cases, molecular collisions with cell walls become more frequent than intermolecular collisions, suppressing the effective thermal conductivity of the gas phase. As the density increases, the foam structure becomes finer and more uniform, resulting in smaller cell sizes. Therefore, this effect helps explain the thermal conductivity in closed-cell materials. In addition to gas conduction, radiative heat transfer also contributes to the total thermal conductivity of EPS, particularly in lower-density foams. Radiation propagates heat transfer across and between cells. In lower-density EPS, the larger cells and thinner walls provide more efficient radiative transfer. As the density increases, the cell size decreases and the proportion of solid polymer increases, which leads to greater absorption and scattering of thermal radiation. This increases the polymer content and reduces radiative transmission though the material, effectively dampening the radiative component of heat transfer.

In support of this physical framework, the statistical analysis of thermal conductivity results yielded exceptionally high $R^{2}$ values when compared with the predictive empirical model defined in EN 13163:2016 [30], which is based solely on density and derived from virgin EPS. This strong agreement, observed even in samples containing up to 50% mechanically recycled EPS, indicates that density remains the dominant parameter governing thermal conductivity, despite the introduction of recycled content and the potential microstructural variability it introduces. The data suggest that the foam’s internal structure including recycled and virgin bead fusion remains sufficiently homogenous at the macroscale to retain the predictive accuracy of models originally developed for virgin materials. As such, the use of standard density-based equations remains valid and reliable for the design and quality control of EPS insulation products containing significant shares of recycled material.

Figure 3a presents a view of the reference EPS with a density of 20 kg/m^3^ representing a typical virgin EPS structure. The image reveals a highly uniform and consistent cellular architecture, characterized by numerous tightly packed beads. This homogenous and well-fused microstructure is characteristic of high-quality EPS, contributing to its excellent thermal insulation properties by effectively trapping air and minimizing heat transfer through convection, conduction, and radiation. The robust fusion between beads also provides superior mechanical integrity, leading to high compressive and bending strengths.

Figure 3b displays the structure of the EPS sample incorporating 25% recycled material. Compared with the virgin reference material, heterogeneity is visible and the recycled material is easily spotted as it is characterized by an irregular shape and size due to mechanical grinding. Despite this visual heterogeneity, the bead fusion remains largely intact, and the overall microstructure appears cohesive. This suggests that, at this level of recycled content, material integrity is maintained, and the modest reduction in mechanical and thermal performance is more closely related to the decrease in density than to defects in bonding or structure.

Figure 3c shows the structure of an EPS sample containing 50% recycled material. In comparison to both the reference sample (Figure 3a) and the 25% recycled sample (Figure 3b), the degradation in structural uniformity becomes more pronounced. The image reveals a highly heterogeneous bead arrangement, where irregularities in the shape, size, and surface texture of the recycled granules are clearly visible. The recycled EPS samples, due to their prior use and mechanical processing, exhibit reduced expandability and altered surface properties. Nevertheless, the fusion between beads remains continuous and functional. The mechanical and thermal property degradation observed in this series are primarily attributed to the density reduction, rather than to any systematic failure in bead cohesion. These findings support the conclusion that, even at high recycled contents, adequate inter bead bonding can be achieved and density remains the dominant factor influencing EPS performance.

An additional observation from the dataset is the increase in standard deviation for both mechanical and thermal properties when the recycled EPS content exceeds 25%. This effect is pronounced in the S2 series, where greater variability is evident in the compressive strength and thermal conductivity results. The higher standard deviation suggests that higher recycled content introduces greater heterogeneity within the material, likely due to the broader distribution of bead sizes and shapes and the partial fusion quality of the recycled granules. These inconsistencies can lead to localized deviations in density and microstructural uniformity, which affect the reproducibility of performance. This trend underscores the importance of tight quality control when using higher volumes of mechanically recycled EPS, especially from post consumer packaging sources.

## 5. Conclusions

This study investigated the mechanical and thermal performance of EPS composites made from virgin EPS and varying proportions of mechanically recycled EPS (5%, 10%, 25%, and 50%). Two sources of mechanically recycled EPS were considered (construction EPS (S1) and packaging EPS (S2)). The tested properties included density, compressive strength at 10% deformation, bending strength, and thermal conductivity. The results demonstrate that the density decreased as the recycled content increased, primarily due to the lower intrinsic density of the mechanically recycled EPS, which was 15–17 kg/m^3^. This decline was more pronounced in samples based on higher-density virgin EPS. Notably, a significant drop in density and mechanical performance became evident when the recycled content exceeded 10% in the S1-25 series and 25% in the other series.

Compressive strength at 10% deformation closely followed trends predicted by EN 13163, where compressive strength is primarily a function of density. Despite the inclusion of recycled material, the majority of data points aligned well with the empirical model for virgin EPS, and only a few samples approached the lower bound of the 95% confidence interval. This suggests that the existing predictive models remain valid even with recycled content up to 50%. Both the S1 and S2 series exhibited similar compressive behavior up to 50% recycled content, after which deviations became more noticeable, particularly in bending strength. The S1 series retained better bending strength than S2, with the S1–25 samples containing 50% recycled content showing a reduction of 42.6% compared with virgin EPS.

Thermal conductivity remained stable at lower recycled content levels. Increases were minor (generally under 5%) up to 25% recycled EPS in both the S1 and S2 series. At 50% recycled content, the thermal conductivity rose modestly (by about 4% to 6% depending on the series), but values remained within the expected variability defined by EN 13163:2016. The origin of the recycled EPS (construction vs. packaging) showed no significant effect on thermal conductivity up to 25% recycled content.

This study confirms that density remains a reliable predictor of compressive strength in EPS composites containing recycled material and that the EN 13163:2016 empirical models can reasonably predict compressive behavior even with significant amounts of recycled material. However, the cause of property degradation at higher recycled levels, likely linked to microstructural factors such as bead fusion quality or porosity, calls for further investigation, ideally supported by microstructural analyses.

A comparison between the experimental results and the predictive models defined in EN 13163:2016 demonstrated high consistency across all tested series. The lowest coefficient of determination $\left(R^{2}\right)$ for compressive stress at 10% deformation was 0.962, observed in sample S2-25-50%. For thermal conductivity, the lowest $R^{2}$ value was 0.997, found in the S2-20-5% and S2-20-10% samples. These results confirm that the empirical models outlined in the standard provide a strong fit even when applied to materials incorporating mechanically recycled EPS. Interestingly, the lowest $R^{2}$ values for mechanical and thermal properties did not occur in the same samples. This divergence may suggest that the underlying mechanisms affecting compressive strength, such as bead bonding quality, internal voids, and recycled bead distribution, do not necessarily impact thermal conductivity in the same way. While density remains a reliable predictor for both properties, this finding underscores the multifactional nature of performance changes introduced by recycled content.

## Figures and Tables

**Table 1:** EPS block information.

Series	Recycled EPS Content (%)	Target Block Density (kg/m^3^)	Type of Recycled EPS
R-20-0%	0	20	Construction (S1)
S1-20-5%	5		
S1-20-10%	10		
S1-20-25%	25		
S1-20-50%	50		
R-25-0%	0	25	Packaging (S2)
S1-25-5%	5		
S1-25-10%	10		
S1-25-25%	25		
S1-25-50%	50		
S2-20-5%	5	20	Construction (S1)
S2-20-10%	10		
S2-20-25%	25		
S2-20-50%	50		
S2-25-5%	5	25	Packaging (S2)
S2-25-10%	10		
S2-25-25%	25		
S2-25-50%	50		

**Table 2:** Test details.

Test Name	ISO Standard	Number of Samples per Block	Specimen Dimensions (mm)
Density	EN ISO 29470:2020 [26]	3	1000 × 500 × 50
Compressive strength at 10% deformation	EN ISO 29469:2022 [27]	15	100 × 100 × 50
Three-point bending test	EN ISO 12089:2013 [28]	9	300 × 150 × 50
Thermal conductivity	EN ISO 12667:2002 [29]	3	300 × 300 × 50

**Table 3:** $R^{2}$ between experimental compressive stress at 10% deformation and the EN 13163:2016 model.

Series	$R^{2}$
R-20-0%	0.990
S1-20-5%	0.987
S1-20-10%	0.986
S1-20-25%	0.990
S1-20-50%	0.975
R-25-0%	1.000
S1-25-5%	1.000
S1-25-10%	0.997
S1-25-25%	0.998
S1-25-50%	0.998
S2-20-5%	0.995
S2-20-10%	0.999
S2-20-25%	0.994
S2-20-50%	0.971
S2-25-5%	0.999
S2-25-10%	0.999
S2-25-25%	0.991
S2-25-50%	0.962

**Table 4:** $R^{2}$ between experimental thermal conductivity and the EN 13163:2016 model.

Series	$R^{2}$
R-20-0%	0.998
S1-20-5%	0.999
S1-20-10%	0.999
S1-20-25%	0.999
S1-20-50%	0.999
R-25-0%	1.000
S1-25-5%	1.000
S1-25-10%	1.000
S1-25-25%	1.000
S1-25-50%	1.000
S2-20-5%	0.997
S2-20-10%	0.997
S2-20-25%	0.998
S2-20-50%	0.999
S2-25-5%	1.000
S2-25-10%	1.000
S2-25-25%	1.000
S2-25-50%	0.999

## Data Availability

The data presented in this study are available on request from the corresponding author due to privacy.
